# Identification of Immunogenic Cell-Death-Related Subtypes and Development of a Prognostic Signature in Gastric Cancer

**DOI:** 10.3390/biom13030528

**Published:** 2023-03-14

**Authors:** Xuejun Gan, Xiaohuan Tang, Ziyu Li

**Affiliations:** Key Laboratory of Carcinogenesis and Translational Research (Ministry of Education), Department of Gastrointestinal Cancer Center, Ward I, Peking University Cancer Hospital & Institute, Beijing 100142, China; ganxuejun@bjmu.edu.cn (X.G.); tangxiaohuan@bjmu.edu.cn (X.T.)

**Keywords:** immunogenic cell death, gastric cancer, tumor microenvironment, prognosis, immunotherapy

## Abstract

Background: Immunogenic cell death (ICD) is considered a promising type of regulated cell death and exerts effects by activating the adaptive immune response, reshaping the tumor environment (TME) and improving therapeutic efficacy. However, the potential roles and prognostic value of ICD-associated genes in gastric cancer (GC) remain unclear. Methods: The RNA expression data and clinical information of 1090 GC patients from six cohorts were collected. Consensus clustering was used to identify three distinct molecular subtypes. Then, a robust prognostic ICD_score for predicting prognosis was built via WGCNA and LASSO Cox regression according to the TCGA cohort, and the predictive capability of the ICD_score in GC patients was validated in the other cohorts. ICD-related immune features were analyzed using a CIBERSORT method and verified by immunofluorescence. Results: We found that ICD-related gene variations were correlated with clinical outcomes, tumor immune microenvironment (TIME) characteristics and treatment response. We then constructed an ICD signature that classifies cases as low- and high-ICD_score groups. The high-ICD_score group indicates unfavorable OS, a more advanced TNM stage, and presents an immune-suppressed phenotype, which has more infiltrations of pro-tumor immune cells, such as macrophages, which was verified by immunofluorescence. In addition, a nomogram containing the ICD_score showed a high predictive accuracy with AUCs of 0.715, 0.731 and 0.8 on Years 1, 3, and 5. Conclusion: We performed the first and synthesis ICD analysis in GC and built a clinical application tool based on the ICD signature, which paved a new path for assessing prognosis and guiding individual treatment.

## 1. Introduction

Gastric cancer (GC) is the fifth most common cause of tumor-related death worldwide [1], especially in China [2]. Despite the great efforts that have been made in the diagnosis, surgery, chemotherapy, and immunotherapy of GC, the recurrence rate and mortality remain high. The platinum-based chemotherapy still remains one of the most important therapeutic strategies for GC treatment. Significantly, oxaliplatin, the third generation of platinum-based drug, has almost replaced cisplatin in the treatment for GC. According to recent studies, the greater efficacy of oxaliplatin is mostly owed to its ability to induce additional immunogenic cell death (ICD) [3,4].

In the complex tumor–immune–stromal microenvironment, antigenicity is insufficient for tumor cells to elicit antitumor immunity because of the lack of co-stimulatory signals, and then dendritic cells cannot effectively deliver antigens to immunocytes, which results in immunological tolerance [5]. Adjuvanticity is a vital factor that promotes the recruitment and activation of antigen-presenting cells. Tumor cells mediate immunomodulatory effects by secreting or surface-exposing molecules that can function as danger signals or adjuvanticity, known as “damage-associated molecular patterns” (DAMPs) [6]. Immunogenic cell death (ICD) represents a functionally distinct cell death type that numerous DAMPs that were exposed actively or released passively during cell death, injured or stressed [7,8]. DAMPs released in the process of ICD include calreticulin (CALR), the high-mobility group box1 (HMGB1), the cytoplasmic protein annexin A1 (ANXA1), the heat-shock proteins (HSPs), and the small metabolite ATP [9]. Emerging evidence has demonstrated that ICD triggered by treatment can initiate antitumor immune responses that augment the effects of conventional antitumor chemotherapy and radiotherapy [10,11]. Targeting ICD-associated DAMPs may provide new insights into the treatment of cancer. 

In addition, emerging evidence has shown that immunotherapy brings fresh hope to GC patients, especially PD-1 and PD-L1 inhibitors [12,13]. A recent clinical trial found that oxaliplatin significantly elevated the efficacy of PD-1 blockade by inducing additional ICD in GC, and suggested that ICD biomarkers may be candidate predictive factors for chemo-immunotherapy [14]. These results indicated a potentially essential role of ICD-related genes in the combination of immunotherapy and chemotherapy in GC treatment. Furthermore, since the Checkmate649 trial [15], chemo-immunotherapy is going to achieve dominance in GC treatment. However, the effects of ICD-related genes in GC have not been well expounded. Thus, a synthesis analysis of the roles and underlying applications of the ICD-related genes in GC may be extraordinarily beneficial.

In the current study, we first analyzed the ICD-related genes to explore the effects of ICD in the tumor microenvironment (TME) and the survival of GC patients. An ICD_score signature was constructed, which can predict the tumor immune microenvironment (TIME), prognosis, and treatment response of GC. Additionally, a nomogram combining the ICD_score signature with clinicopathological features increased the power for predicting clinical outcomes and treatment responses. All the efforts may help the clinical treatment decision-making process for GC in the future.

## 2. Materials and Methods

### 2.1. Data Collection

RNA sequencing (RNA-seq) expression data, somatic mutation data, copy number variation data and the clinical information of GC patients were downloaded from the cancer genome atlas (TCGA) database (https://cancergenome.nih.gov/ (accessed on 15 November 2022)). The expression data were normalized utilizing the “limma” R package. Microarray datasets were obtained from the gene expression omnibus (GEO) databases (http://www.ncbi.nlm.nih.gov/geo/ (accessed on 10 November 2022)). Four datasets based on the GPL570 platform were integrated as a combined GEO cohort applied as the external validation cohort, including GSE15459 [16], GSE34942 [17], GSE57303 [18], GSE62254 (ACRG) [19]. The batch effects among the four datasets were corrected by using the “Combat” algorithm. Next, we enrolled 607 GC patients in our subsequent analyses due to patients whose overall survival was less than 1 month being excluded. Immunotherapy datasets were obtained from IMvigor210 whose patients were being treated with anti-PDL1 therapy [20]. Additionally, another cohort of 198 patients from our center, the Peking University Cancer Hospital (PKUCH, Beijing, China), was used for validation, and another 35 patients from our center who underwent standard perioperative chemotherapies were utilized to examine the predictive power of the ICD_score signature in response to chemotherapy. This study was approved by the Ethical Committee of PKUCH and the above patients from PKUCH signed informed consent. Table S4 summarized the details of the GC datasets used in this study.

### 2.2. Identification of ICD-Related Subgroups by Consensus Clustering

First, 53 ICD genes were extracted from previous publications, and their information is presented in Table S1. According to ICD-associated gene expression, we utilized an unsupervised consensus clustering analysis provided by the “ConsensusClusterPlus” R package to stratify patients into disparate subtypes. We evaluated the appropriate cluster numbers k from 2 to 10, and the process was replicated 1000 times to guarantee the stability of the results. Subsequently, Kaplan–Meier survival analysis were performed to evaluate the differences in clinical outcomes in different clusters via utilizing the “survival” and “survminer” R packages. 

### 2.3. Robust DEGs Identification and Functional Enrichment Analysis

The differentially expressed genes (DEGs) among the different ICD-associated clusters were characterized with inclusion standard (|fold-change| > 1, adjusted *p* < 0.05) by utilizing the “limma” R package. To further investigate the functional differences of the ICD pattern and determine different signal pathway and biological effects, we executed gene ontology (GO) and Kyoto Encyclopedia of Genes and Genomes (KEGG) analyses according to the DEGs utilizing the “clusterProfiler” R package, with a screening threshold of (*p* < 0.05) [21]. 

### 2.4. Characterization of TME and Immune Infiltration among Three ICD Subtypes

To exhibit the immune characteristics of GC samples, the CIBERSORT algorithm was conducted to assess the abundance of 22 immune infiltrating cells [22]. We also analyzed the expression of PD-1, PD-L1, and CTLA4 among the three ICD-related subtypes. In addition, we evaluated the stromal and immune scores of each patient through the estimation of stromal and immune cells in malignant tumor tissues using expression data (ESTIMATE) algorithm [23]. 

### 2.5. Co-Expression Network Construction and Hub Genes Identification

Weighted gene co-expression network analysis (WGCNA) is a widely applied method to transform the gene expression matrix into unsigned co-expression networks and then uncover critical interacted gene modules and hub genes [24]. Here, we used the “WGCNA” R package to construct a co-expression network based on the above extracted 1177 dysregulated DEGs from the TCGA cohort. We first performed hierarchical clustering analysis on the DEG expression through the “goodSamplesGenes” function and removed outlier genes and samples. To test the average connectivity degree of different modules and their independence, candidate power values from 1 to 20 were applied, and then the appropriate power value was determined when the degree of independence was over 0.9 by utilizing the “pickSoftThreshold” function. After transforming the adjacency matrix into the topological overlap matrix (TOM), we calculated the TOM and the TOM-based dissimilarities, and then sorted these genes into different modules. Subsequently, the dynamic tree-cutting algorithm was applied to build a cluster dendrogram for the identification of modules. The module with the highest related coefficient was selected for further exploration. Finally, the union of candidate hub genes was defined as hub genes, and then we performed GO analyses on these genes via clueGO, a plug-in of Cytoscape. 

### 2.6. Construction and Validation of the ICD-Associated Prognostic ICD_Score

A score of ICD-related risk was measured to quantify the individual tumors’ ICD-associated patterns and predict patient prognosis. First, the hub genes and ICD-related genes were included in a subsequent univariate Cox regression analysis, and we set 0.05 as the cut-off *p*-value. After that, the GC patients of TCGA-STAD cohort were randomly classified into training and testing sets with a proportion of 1:1; then, we used the training group to build the ICD-associated prognostic ICD_score by the LASSO Cox regression model (“glmnet” R package) according to prognostic genes. Ultimately, we kept 3 genes and their coefficients by selecting the optimal penalty parameter (λ value) with the minimum criteria. The ICD_score was calculated as follows: ICD_score= $\sum_{i}^{3}\left(EXPi*Coefi\right)$ (*EXP*: gene expression level, *Coef*: risk coefficients). According to the median ICD_score, patients in the training group were classified into low (ICD_score < median) and high (ICD_score > median) ICD_score groups. Next, the Kaplan–Meier survival analysis and principal component analysis (PCA) based on the low- and high-ICD_score groups were carried out. The 1-, 3-, and 5-year receiver operating characteristic (ROC) curve analyses were conducted using the “survival”, “survminer” and “timeROC” R packages. Similarly, the testing and external validation cohort (a combined GEO cohort, *n* = 607) were also stratified into low- and high-ICD_score groups by applying the cut-off value from the training cohort and performing the same Kaplan–Meier survival analysis and ROC curves analysis. 

### 2.7. Clinical Correlation of the ICD_Score Signature

The clinical information (age, gender, grade, T category, N category, M category, and TNM stage) were obtained from the TCGA cohort. We used chi-square tests to investigate the ICD_scores’ relations to the clinical traits. To evaluate the characterization of immune landscape, CIBRSORT was applied to quantify the relationships between the abundance of infiltrating immune cells and the ICD_score. Furthermore, we examined the differences in the expression of immune checkpoints between the low- and high-ICD_score groups.

### 2.8. Gene Set Variation Analysis (GSVA)

Gene set variation set (GSVA) was performed to investigate the differences between the two ICD_score groups in signal pathways based on the hallmark gene set (h.all.v7.2) extracted from MSigDB database by using “GSVA” R package [25]. A *p* < 0.05 was considered as statistically significant between distinct subgroups.

### 2.9. Assessment of Tumor Regression Grade (TRG) 

After undergoing neoadjuvant chemotherapy, the degree of tumor regression was evaluated according to the National Comprehensive Cancer Network (NCCN) guidelines (www.nccn.org/patients, accessed on 1 December 2022): Grade 0, no residual tumor cells, which means complete regression; Grade 1, minimal residual cancer with single cells or rare groups of cancer cells, which means near-complete response; Grade 2, residual cancer with evident tumor regression, but more than single cells or small groups of cancer cells also known as partial tumor regression; Grade 3, extensive residual cancer with no evident tumor regression, which indicates poor or no response. We categorized patients with TRG 0 or 1 as responders, while patients with TRG 2 or 3 as non-responders.

### 2.10. Multiplex Immunofluorescence (mIF)

Multiplex immunofluorescence staining was performed with the Akoya OPAL Polaris Automation IHC kit (NEL871001KT). Briefly, FFPE tissue sections were dewaxed in a BOND RX system and then incubated with primary antibodies sequentially targeting CD68 (1:1000, ab213363, Abcam, Cambridge, MA, USA), CD163 (1:500, Abcam, ab182422), and CD56 (1:1000, Abcam, ab75813). These slides were then incubated for 1 h at room temperature with special secondary antibodies, followed by reactive Opal fluorophores. The nuclei acids were stained using DAPI. The tissue sections without fluorophores were used as negative controls. Multiplex-stained slides were scanned at 20-nanometer wavelength intervals from 440 nm to 780 nm, using A Vectra Polaris Quantitative Pathology Imaging System (Akoya Biosciences, MA, USA) and analyzed quantitatively by inForm v.2.4.8 (Akoya Biosciences). The quantities of various cell populations were determined as the percentage of positively stained cells in all nucleated cells per square millimeter.

### 2.11. Establishment and Validation of a Nomogram Scoring System

First, univariate analysis was conducted with clinicopathological features and ICD_score in the TCGA cohort. According to the results of univariate analysis, the significant factors (*p* < 0.05) were subsequently used as an input to develop a predictive nomogram by utilizing the “rms” R package [26]. The predictive accuracy of the nomogram scoring system was evaluated by ROC analyses for 1-, 3-, and 5-year survivals. Calibration curves were applied to depict consistency between the predicted survival events and the actual observations. 

### 2.12. Statistical Analyses

Student’s *t*-test was utilized for comparisons of continuous variables between two groups, and the chi-square test was utilized to compare the categorical variables. We applied the Kaplan–Meier method to compare the OS of patients between subgroups. We used univariate and multivariate Cox regression models to evaluate the independent prognostic value of the signature. A *p* ≤ 0.05 was considered a statistical significance. All statistical analyses were accomplished using R software (v4.0.2).

## 3. Results

### 3.1. Landscape of Genetic and Transcriptional Alterations of ICD-Related Genes

**Figure 1:**
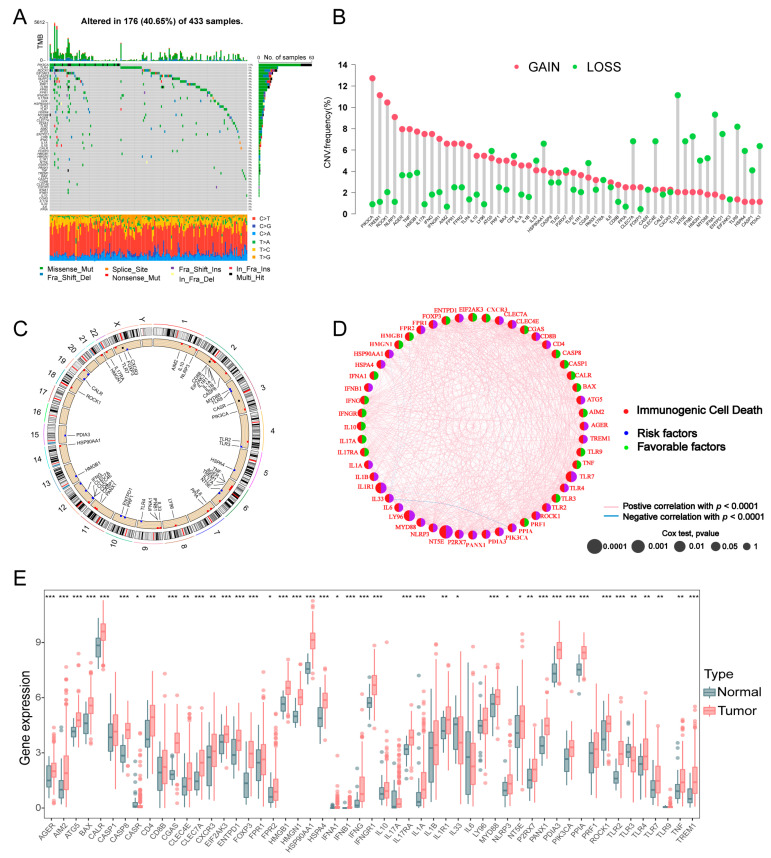
Genetic and transcriptional variations of ICD genes in GC. (**A**) Mutation frequencies of 53 ICD genes in GC patients from the TCGA cohort. (**B**) Frequencies of CNV gain and loss in ICD genes. (**C**) Locations of CNV alterations of ICD genes on 23 chromosomes. (**D**) Interactions and prognostic value of ICD-related genes in GC. (**E**) Differences in expression distributions of 53 ICD genes between GC and normal tissues (tumor, red; normal, grey). *p* values were shown as: * *p* < 0.05; ** *p* < 0.01; *** *p* < 0.001. ICD, immunogenic cell death; GC, gastric cancer; TCGA, the Cancer Genome Atlas; CNV, copy number variation; OS, overall survival.

The flowchart of the current study is presented in Figure S1. First, 53 ICD-associated genes were identified and collected from previous studies [7,27,28,29,30] and their detailed information was exhibited in Table S1. To fully understand the roles of the ICD-related genes involved in tumorigenesis, 433 patients from the TCGA-STAD cohort and 607 patients from a GEO-integrated cohort based on the GPL570 platform (including GSE15459, GSE34942, GSE57303, and GSE62254) were enrolled in our study. We analyzed the frequencies of the somatic mutations of the 53 ICD-related genes and the results showed a 40.65% mutation frequency in the TCGA cohort (Figure 1A). Among them, the mutation frequency of PIK3CA (15%) was the highest, followed by TLR4, ROCK1, and EIF2AK3. Subsequently, we examined the copy number alterations (CNVs) of 53 ICD-related genes in GC. Among them, PIK3CA exhibited the highest amplification frequency, while TLR3, TLR9, IFNA1, and ENTPD1 had widespread CNV loss (Figure 1B). Figure 1C showed the locations of the CNVs and CNV frequency in the ICD-related genes. As the PIK3CA had the highest frequency of alteration and amplification, the correlation between PIK3CA mutation and ICD-related gene expression was explored. The results revealed that the expression levels of approximately 50% of ICD-related genes were correlated with the status of the PIK3CA mutation (Figure S2A). 

Simultaneously, we investigated the mRNA expression patterns of the ICD genes in GC tissues and the corresponding normal tissue samples. Intriguingly, 49 (92.4%) genes were upregulated in tumor tissues, while TRL3, which bore the highest frequency of CNV loss, was expressed at a lower level, significantly in GC samples compared to normal samples (Figure 1E), suggesting that CNVs alterations affected the ICD-related genes expression. However, some ICD-related genes with CNVs loss also increased mRNA expression, such as CLEC7A, ENTPD1, and PDIA3. CNVs alterations are a factor that could account for the partial changes of the ICD gene expression, but it is not the only factor that regulates gene expression. Furthermore, we used univariate Cox regression and Kaplan–Meier analysis to investigate the prognostic value of ICD-related genes and we found that four genes (IL1R1, LY96, NT5E and TLR7) were identified as prognostic factors (Table S2). As shown in Figure 1D, the interactions and regulator connections of ICD-related genes in GC patients were comprehensively analyzed.

### 3.2. Identification of ICD-Associated Subtypes in GC

**Figure 2:**
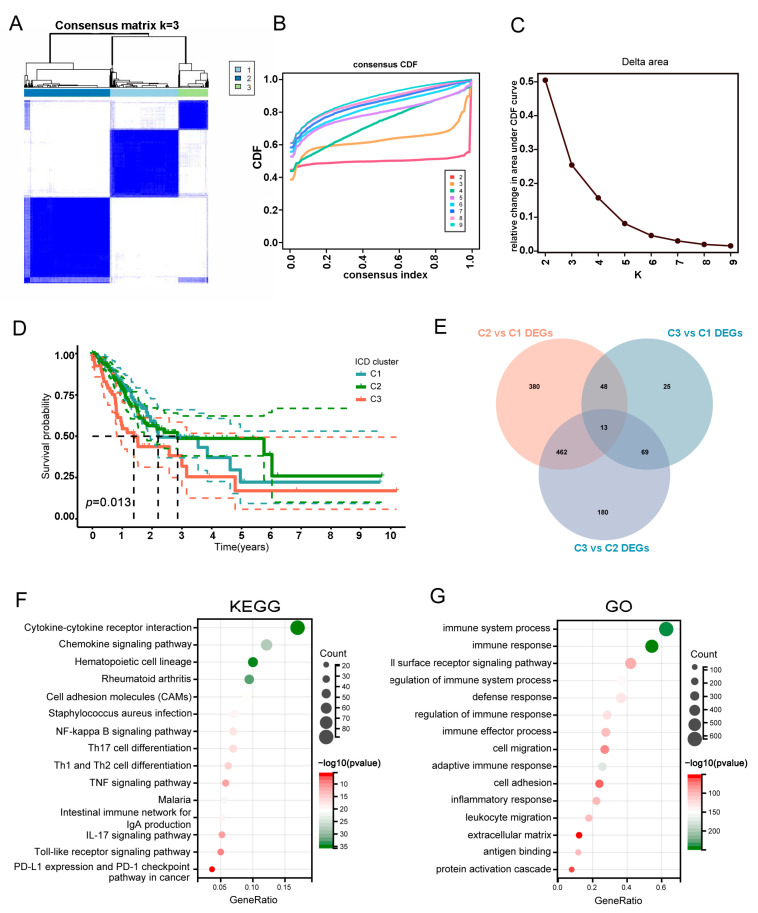
Identification of ICD-related subgroups by consensus clustering. (**A**–**C**) Consensus matrix heatmap defining 3 clusters (k = 3). (**D**) Kaplan–Meier curves of OS in distinct subtypes. (**E**) Venn diagram showing 1177 DEGs between the three ICD-related subtypes. (**F**,**G**) KEGG and GO enrichment analyses of DEGs among three ICD-related subtypes. DEGs, differentially expressed genes; KEGG, Kyoto Encyclopedia of Genes and Genomes; GO, gene ontology.

To further investigate the expression patterns of the ICD-associated genes, we categorized the GC patients derived from the TCGA cohort using a consensus clustering algorithm. We identified the optimal clustering stability at k = 3 for sorting the TCGA cohort into three clusters, which included Cluster1 (C1, *n* = 137), Cluster2 (C2, *n* = 174), and Cluster3 (C3, *n* = 57) (Figure 2A–C). The Kaplan–Meier (KM) curve analysis revealed that patients in the C2 subtype possessed a significantly prolonged OS, while patients in the C3 subtype had the worst outcome (*p* = 0.013, Figure 2D). 

To investigate the underlying biological behaviors of each ICD-associated subtype, we analyzed the significant DEGs and enriched signaling pathways in distinct subtypes. Here, a total of 1177 dysregulated genes were detected among the three subtypes (Figure 2E). As expected, KEGG pathway analyses showed that the DEGs were enriched in the cytokine–cytokine receptor interaction significantly, which is the main way for DAMPs to exert their effects [31,32,33,34], Th1/Th2 cell differentiation, and TNF signaling pathway, as well as NF-kappa B signaling pathway (Figure 2F). Consistently, many immunity-associated biological processes also showed significant enrichment in the GO analyses (Figure 2G). Thus, different ICD-related subtypes could cause distinguished remodeling immune microenvironments. 

### 3.3. Characteristics of TIME in Distintct Subtypes

**Figure 3:**
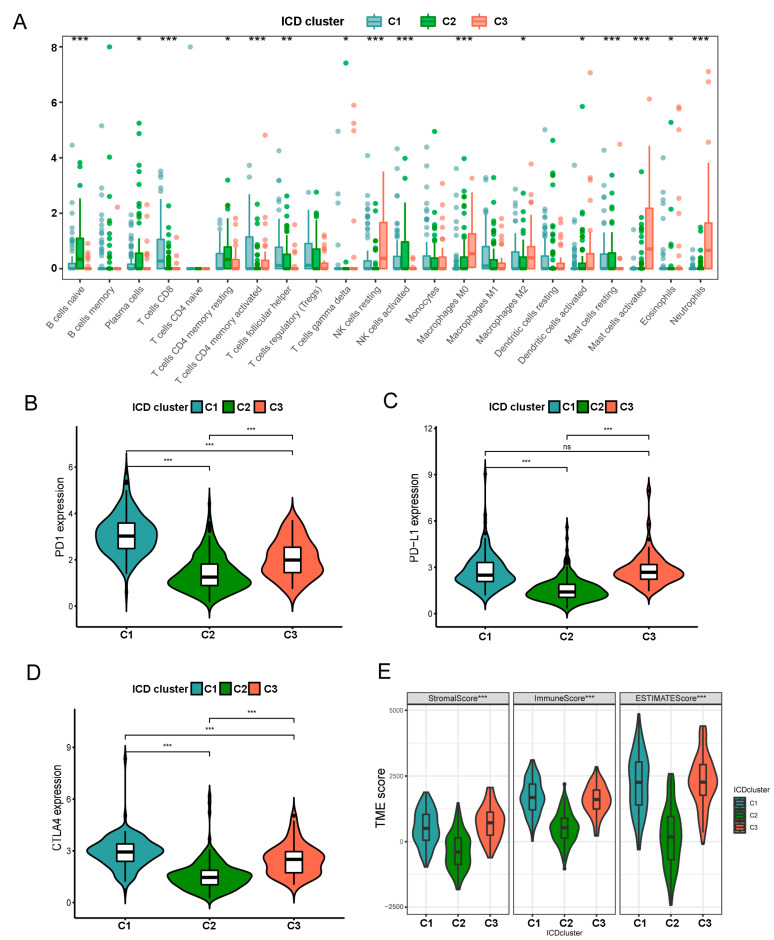
(**A**) The abundance of 22 infiltrating immune cell types between the three ICD-related clusters were analyzed by CIBERSORT in TCGA cohort. (**B**–**D**) Differential expression of PD-1, PD-L1, and CTLA4 in the three ICD-associated subtypes. (**E**) Correlations between the three ICD clusters and TME score. *p* values were shown as: * *p* < 0.05; ** *p* < 0.01; *** *p* < 0.001. TIME, tumor immune microenvironment; TME, tumor microenvironment.

According to the functional analyses, we further evaluated the correlations between the three ICD-associated subtypes and 22 immune cell subsets identified by the CIBERSORT algorithm [22]. Surprisingly, we found obvious differences in the proportion of the tumor-infiltrating immune cells in distinct subtypes (Figure 3A). Activated NK cells in C2 had the highest infiltration level, while M2 macrophages and regulatory T cells (Tregs) infiltrated significantly lower in C2 than the other two subtypes. Moreover, patients with C3 subtype exhibited considerably elevated percentages of resting NK cells, activated mast cells, M0 and M2 macrophages and neutrophils. Similarly, analyses of three important immune checkpoints, including PD-1, PD-L1, CTLA4, revealed lower expression in C2, which indicates a lower level of immunosuppression (Figure 3B–D). Then, we assessed the TME scores (stromal score, immune score, and estimate score) of the three subtypes (Figure 3E). For the TME scores, increasing the ImmuneScore or StromalScore indicated that the TME contained more immune or stromal cells. These results indicated that patients in the C2 subset had more antitumor compounds, which might contribute to their longer survival time.

### 3.4. Construction of the Prognostic ICD_Score Signature

**Figure 4:**
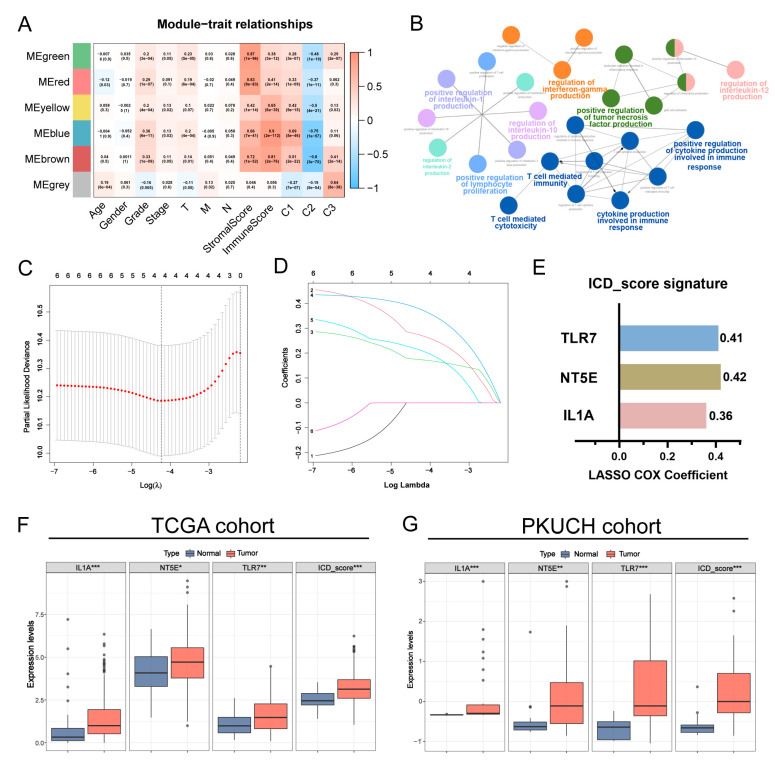
Identification of hub genes by WGCNA and construction of the prognostic ICD_score. (**A**) Heatmap of the correlation between module eigengenes and clinical traits of GC. Each cell includes Pearson correlation coefficient and *p* value, respectively. (**B**) Functions and pathways of the hub genes were computed and visualized using clueGO. (**C**,**D**) 10-time cross validation for tuning parameter selection by LASSO regression. The solid vertical lines are partial likelihood deviance ± standard error (SE). The dotted vertical lines are drawn at the optimal values by minimum criteria and 1-SE criteria. (**E**) The ICD_score signature construction. (**F**,**G**) Expression levels of 3 selected genes and calculated ICD_score between tumors and peritumor tissues in TCGA cohort and our PKUCH cohort, respectively. *p* values were shown as: * *p* < 0.05; ** *p* < 0.01; *** *p* < 0.001. WGCNA, weighted gene co-expression network analysis; TOM, topological overlap matrix; GS, gene significance; MM, module membership; LASSO, least absolute shrinkage and selection operator regression.

To identify the key modules most correlated with the ICD-associated subtypes and clinical features, we conducted a WGCNA analysis on the TCGA cohort incorporating the DEGs derived from the above analyses. Clinical information was shown in Table S3. After the soft thresholding value was set as 10 (R^2^ = 0.96) and cut height was 0.15, we identified six co-expression modules eventually (Figure 4A; Figure S3A–C). According to the heatmap of module–trait relationships, blue and brown modules revealed higher correlations with clinical traits (Figure S3D–I). In detail, R^2^ = 0.9 and *p* = 2 × 10^−112^ with ImmuneScore, R^2^ = 0.36 and *p* = 6 × 10^−11^ with tumor grade, and R^2^ = 0.69 and *p* = 5× 10^−46^ with C1 in blue module (Figure S3D–F). In brown module, R^2^ = 0.81 and *p* = 2× 10^−76^ with ImmuneScore, R^2^ = 0.33 and *p* = 1× 10^−9^ with tumor grade, and R^2^ = 0.51 and *p* = 2× 10^−22^ with C1 (Figure S3G–I). Herein, we determined blue and brown modules as the key modules. Hub genes were defined as the union of the blue module and brown module. A total of 244 genes were selected as candidate hub genes according to the threshold standard (Module Membership > 0.8 and Gene Significance > 0.5) (Figure S3J). We then investigated the potential biological role of these genes via clueGO, a plug-in of Cytoscape that can be used to classify and visualize GO terms as networks [35]. Based on *p* < 0.05, twenty-eight significant GO terms for the biological process are shown in Table S5. The highest number of candidate genes was classified directly under the regulation of chemokine production, such as interleukin and interferon, followed by the regulation of T cell proliferation and T-cell-mediated immunity (Figure 4B). 

Next, the hub genes mentioned above plus fifty-three ICD-related genes were utilized to establish a prognostic ICD_score signature by LASSO Cox regression analysis. First, patients in the TCGA cohort were randomly divided into training (*n* = 185) and testing (*n* = 185) groups at a ratio of 1:1, and another integrated GEO dataset (*n* = 607) was served as the external validation set. The six genes (FPR2, IL1A, LY96, NT5E, TLR7, and ST8SIA4) were retained for further investigation through the first screening OS-related genes, utilizing univariate regression analysis (*p* < 0.05 and HRs > 1; Figure S4A), which was followed by LASSO Cox regression analysis. We finally obtained three genes (IL1A, NT5E, and TLR7) with their respective coefficients to construct an ICD-associated predictive signature (ICD_score) (Figure 4C–E). The expression levels of these genes, as well as the calculated ICD_score were significantly increased in the GC samples compared to the normal samples in the TCGA cohort (Figure 4F). Moreover, we compared the mRNA expression levels of the genes constructing the ICD_score in the GC tissues and normal tissues of GC patients in our cohort. As is shown in Figure 4G, both the mRNA expression of the three genes and the calculated ICD_score were significantly higher in the GC tissues. 

### 3.5. Validation of the ICD_Score Prognostic Signature

**Figure 5:**
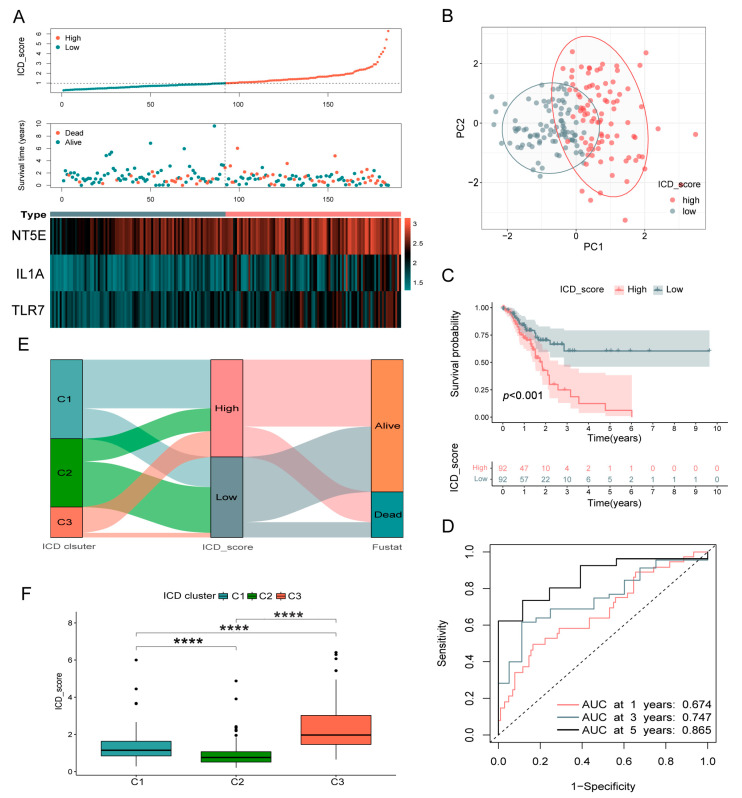
Validation of the ICD_score signature. (**A**) Risk score distribution, survival status of each patient, and heatmaps of prognostic three gene signature in TCGA database. (**B**) Principal component analysis based on ICD_score signature genes to distinguish low and high ICD_score subgroups in TCGA cohort. (**C**) Kaplan–Meier analysis of the OS between the two groups. (**D**) ROC curves to predict the sensitivity and specificity of 1-, 3-, and 5-year survival according to the ICD_score. (**E**) Alluvial diagram of subtype distributions in groups with different ICD_scores and survival outcomes. (**F**) Differences of ICD_score among ICD-related subtypes. ROC, receiver operating characteristic. **** *p* < 0.0001.

To confirm the prognostic value of the constructed ICD_score signature, we analyzed the association between patients’ survival and the signature. In order to increase the accuracy, we utilized the median value as the cut-off point. Then, the correlative analyses were first performed in the training set. The distribution curve of the ICD_scores showed that with the increase in the ICD_scores, survival times shortened significantly (Figure 5A). In addition, the PCA analysis verified remarkable distribution differences between the low- and high-ICD_score groups according to the ICD-scores (Figure 5B). Kaplan–Meier survival curves suggested that patients with high ICD_scores had a significantly worse OS than patients with low scores (*p* < 0.001; Figure 5C). Based on ROC analysis, the AUCs of the 1-, 3-, and 5-year OS were 0.674, 0.747, and 0.865, respectively (Figure 5D). Moreover, we observed remarkably different ICD_scores in disparate ICD-related subtypes. Figure 5E depicts the distribution of patients in the three ICD-related clusters and two different survival states of patients. The ICD_score from the C2 group was the lowest, while that of C3 was the highest, which was consistent with the above conclusions (Figure 5F). 

To further verify the prognostic role of the ICD_score, we applied it to both the internal validation group (testing set from TCGA cohort) and external validation group (a GEO-integrated cohort based on the GPL570 platform including GSE15459, GSE34942, GSE57303, and GSE62254). Using the same cut-off value as the training set, the patients were also assigned to low- or high-ICD_score group. The results of the KM survival analysis, patient score distribution plot, and PCA analysis in validation groups showed similar tendencies to the results of the training group (Figure S5A–F), indicating that the ICD_score signature exhibited a generalized prognostic ability in GC patients.

### 3.6. Clinicopathologic Characteristics in Low- and High-ICD_Score Groups

**Figure 6:**
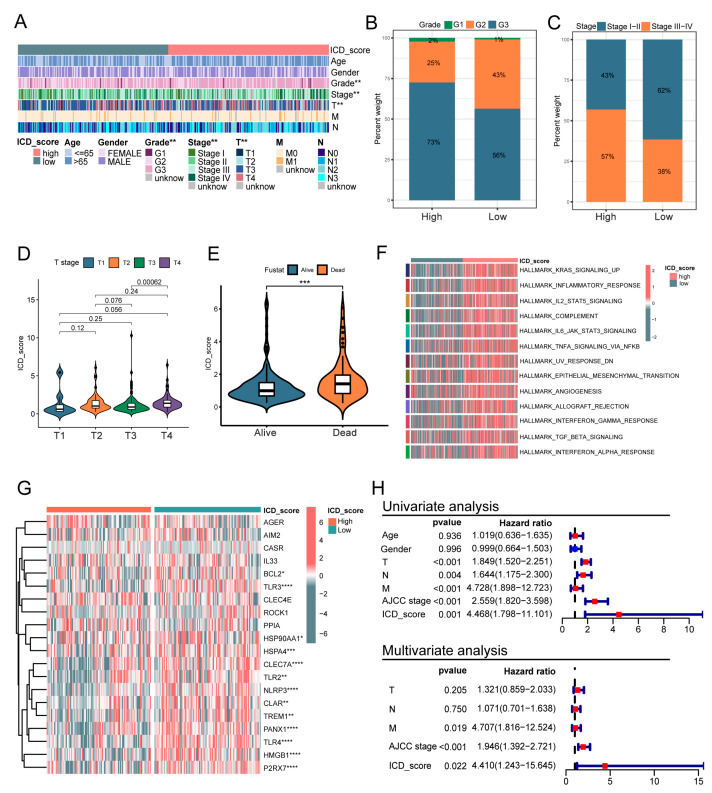
Comprehensive analysis of the ICD_score in GC. (**A**) Relationships between clinicopathologic features and the two ICD_score groups. (**B**,**C**) Correlations between ICD_score and tumor grade and stage in TCGA cohort, respectively. (**D**) ICD_scores of patients with different T-category in TCGA cohort. (**E**) ICD_scores of GC patients with different survival statuses. (**F**) GSVA of hallmark pathways between two distinct ICD_score groups, in which red and grey represent activated and inhibited pathways, respectively. (**G**) Expression pattern of DAMPs-related genes in different ICD_score group. (**H**) Univariate and multivariate Cox regression analysis regarding OS in our cohort. *p* values were shown as: * *p* < 0.05; ** *p* < 0.01; *** *p* < 0.001; **** *p* < 0.0001. GSVA, gene set variation analysis; DAMPs, damage-associated molecular patterns.

Next, we analyzed the correlation between ICD_score and different clinical traits including age, gender, grade, T stage, N stage, M stage, and TNM stage (Figure 6A). There were significant differences in tumor grade, TNM stage, and T-category between the low- and high-ICD_score groups. Patients with high ICD_scores harbored more high-grade tumors (G3), which means more low-differentiation tumor cells than in the patients in the low-ICD_score group (73% vs. 56% in the TCGA cohort, Figure 6B). In the high- ICD_score group, a higher TNM stage was observed compared to the low- ICD_score group (57% vs. 38% in the TCGA cohort; Figure 6C). Additionally, a higher ICD_score was correlated with an increased T stage (Figure 6D). In accordance with the above, it was observed that high ICD_score patients were at higher risk of death (Figure 6E). GSVA enrichment analysis showed that the high-ICD_score group was significantly enriched in tumorigenic signaling pathways, including KRAS signaling up, IL2_STAT5 signaling, epithelial mesenchymal transition and angiogenesis (Figure 6F). In addition, we compared the expression of DAMPs between low- and high-ICD_score groups in our cohort, which included 198 GC patients. As expected, the results showed that most of the DAMPs-related genes were upregulated in the low-ICD_score group, which suggested an immune-activating anti-tumor microenvironment (Figure 6G). Then, the univariate and multivariate Cox regression analyses were performed to confirm the prognostic value of the ICD_score signature in our cohort, and the results showed that the ICD_score was an independent prognostic factor (*p* = 0.022, HR: 4.410, 95%CI: 1.243–15.645) for OS (Figure 6H).

### 3.7. The Immune Landscape between High- and Low-ICD_Score GC

**Figure 7:**
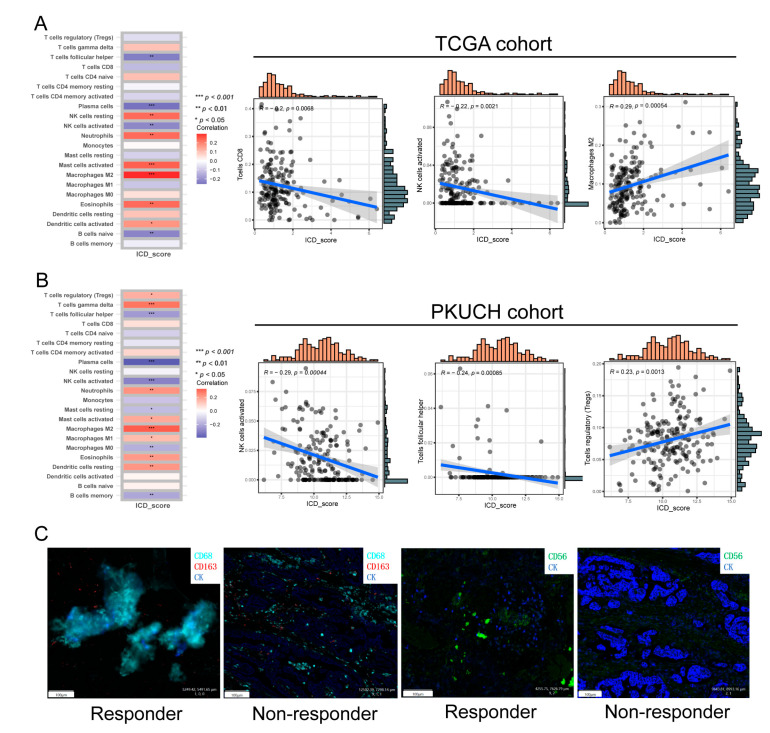
Evolution of the TIME in distinct ICD_score groups. (**A**,**B**) Correlations between ICD_score and immune cell types in TCGA cohort and our PKUCH cohort, respectively. (**C**) Representative mIF-staining pictures of responder/low- and non-responder/high-ICD_score tumors to neoadjuvant chemotherapy plus PD-1 blockade. *p* values were shown as: * *p* < 0.05; ** *p* < 0.01; *** *p* < 0.001.

To elucidate the immune landscape in the different ICD_score groups, we investigated the relationship of the ICD_score signature with the abundance of immune cells, which was calculated by the CIBERSORT algorithm. As depicted in Figure 7A, the ICD_score was positively correlated with the M2 macrophages, activated mast cells, and neutrophils, while it was negatively correlated with CD8 + T cells, activated NK cells, and follicular helper T cells in TCGA cohort. Likewise, in our cohort, with the increased ICD_score, a higher abundance of the immunosuppressive cells (regulatory T cells and M2 macrophages) and a lower abundance of anti-tumor immunocytes (activated NK cells and follicular helper T cells) were observed (Figure 7B).

To validate the association between the ICD_score and TIME, we performed mIF on the resected GC samples of the responder and non-responder to neoadjuvant chemotherapy plus a PD-1 blockade. Consistent with the results of sequencing, responders/low ICD_score had a higher infiltration rate of anti-tumor immune subsets, including an increased M1/M2 macrophage ratio and more infiltrated NK cells (Figure 7C). 

### 3.8. Correlation between ICD_Score and Treatment Response

**Figure 8:**
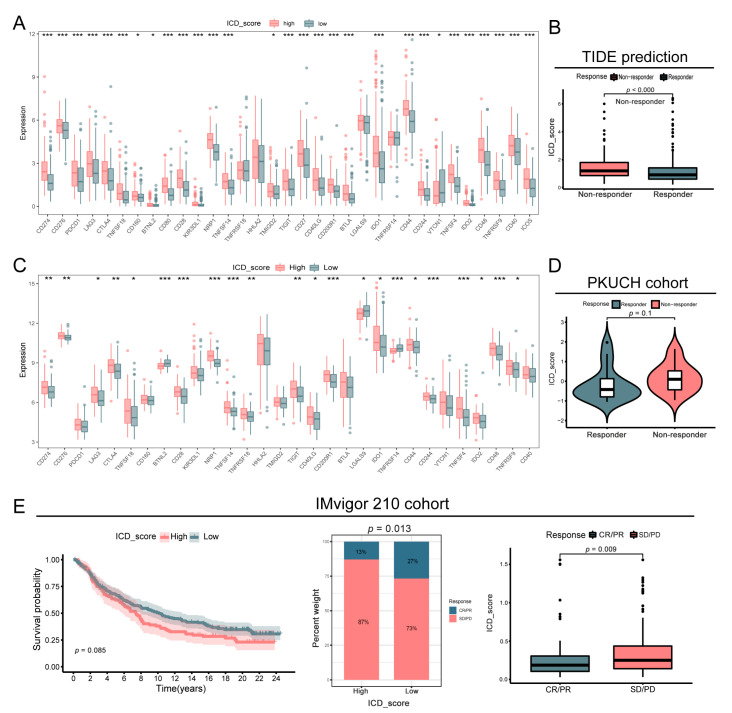
Association of ICD_csore with response to treatment. (**A**) Expression of immune checkpoints in the high- and low-ICD_score groups in TCGA cohort. (**B**) TIDE analysis showed the difference in predicted TIDE scores between two ICD_score patterns in TCGA cohort. (**C**) Expression of immune checkpoints in the high- and low-ICD_score groups from our PKUCH cohort (*n* = 198). (**D**) The differences of ICD_score in responders and non-responders to chemotherapy in a PKUCH cohort (*n* = 35). (**E**) Association of ICD_score with immunotherapy response in IMvigor210 cohort. *p* values were shown as: * *p* < 0.05; ** *p* < 0.01; *** *p* < 0.001. CR, complete response; PR, partial response; SD, stable disease; PD, progressive disease.

Given that immune checkpoints blockade (ICB) has shown promising results in the treatment of GC [36,37,38], here, we explore the correlations between important immune checkpoints and our ICD_score signature. In the TCGA cohort, most of the immune checkpoints were upregulated in the high-ICD_score group, including CD274, PDCD1, CTLA4, IDO1, and so forth, representing an inertia TIME (Figure 8A). Additionally, we applicated a TIDE analysis in the TCGA cohort to assess the clinical predictive role of the ICD_score signature for immunotherapy. The results revealed that compared with the responders, the ICD_score was significantly upregulated in the non-responder group, indicating that immunotherapy could be more beneficial to patients with low ICD_scores (*p* < 0.001, Figure 8B). The same results were found in our validation cohort, which showed that these immune checkpoints were expressed at a higher level in the high-ICD_score group, and that the responders to neoadjuvant chemotherapy had lower ICD_scores compared to non-responders from our cohort (Figure 8C,D).

Additionally, to excavate the performance of the ICD_score on predicting the treatment outcome, we classified patients who received immunotherapy into low- or high-ICD_score group. In the IMvigor210 cohort, patients with low ICD_scores exhibited an immunotherapeutic advantage (*p* = 0.087), and the ICD_score was significantly higher in patients with SD/PD (*p* = 0.013, Figure 8E). Collectively, the reference value of the ICD_score signature for GC treatment, especially immunotherapy, was indicated by our multi-cohort analysis.

### 3.9. Development of a Nomogram to Predict Survival

**Figure 9:**
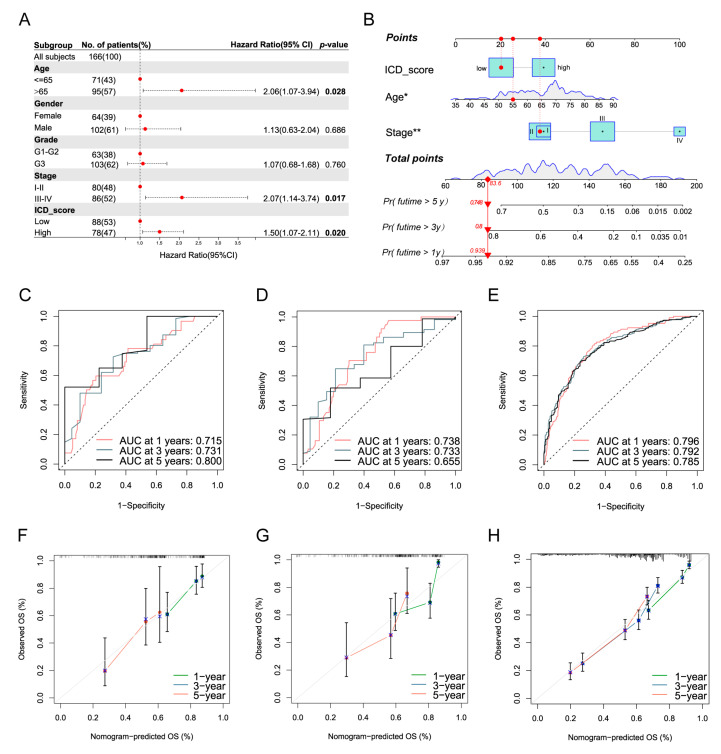
Construction of a nomogram predicting OS based on the ICD_score system in GC. (**A**) Multivariate analysis in the TCGA cohort. (**B**) Nomogram integrated the ICD_score system, age, and TNM stage for predicting the 1-, 3-, and 5-year OS of GC patients in the training set. (**C**–**E**) ROC curves for predicting the 1-, 3-, and 5-year OS in the training, testing, and external validation sets. (**F**–**H**) Calibration curves of the nomogram for predicting of 1-, 3-, and 5-year OS in the training, testing, and external validation sets. *p* values were shown as: * *p* < 0.05; ** *p* < 0.01.

As mentioned above, the KM curve analysis demonstrated that patients with different ICD_scores revealed distinct prognoses (Figure 5C). Here, we performed univariate and multivariate analyses on the ICD_score signature companied by clinicopathological parameters to establish a nomogram scoring system. By calculating the ICD_score of each patient, patients with high ICD_scores revealed significantly shortened OS (high-risk: HR = 1.50, *p* = 0.02) in multivariate analysis compared to those with low ICD_scores (Figure 9A). Finally, we incorporated these independent prognostic parameters, including age, TNM stage, and ICD_score, to build the nomogram, which is capable of predicting the survival probabilities of GC on Years 1, 3, and 5 (Figure 9B). The AUCs for OS on Years 1, 3, and 5 according to the nomogram model indicated a high prognostic validity in the training set, internal testing set, and external validation sets (Figure 9C–E). The ROC analysis implied that the AUCs of the nomogram were 0.715, 0.731 and 0.800 on Years 1, 3 and 5 in the training set, respectively (Figure 9C). In the testing set, the AUCs of 1-, 3-, and 5-year OS were 0.738, 0.733, and 0.655, respectively (Figure 9D). In addition, the AUC values for 1-, 3- and 5-year OS were 0.796, 0.792, and 0.785 in the external validation set (GEO-integrated cohort including GSE15459, GSE34942, GSE57303, and GSE62254; Figure 9E). To further evaluate the predictive ability of the nomogram, we conducted the C-index and ROC analysis to compare the integrated nomogram with age and TNM stage in the training set, internal testing set, and external validation set. Our results exhibited that compared with the age or TNM stage alone, the nomogram model had better survival prediction power (Figure S6A–I). The calibration curves exhibited that the 1-, 3- and 5-year OS predictions by the nomogram were highly consistent with the actual observation not only in the training set, but also the testing and validation sets (Figure 9F–H).

## 4. Discussion

The accumulated evidence indicates that the level of ICD signaling was correlated with the response to treatment and prognosis of patients with GC [39,40,41]. However, most previous studies focused on a single ICD-related gene, which could cause limited prognostic and predictive power, and the overall effects exerted by the ICD-related genes have not yet been fully illuminated in GC. In this study, we first depicted global variations in ICD-related genes at the genetic and transcriptional levels in GC. Three distinct molecular subtypes based on 53 ICD-associated genes were identified. The biological pathways, the characteristics of the TME, and the infiltration of the immune cells have significant differences among the three subtypes. Furthermore, a robust prognostic ICD_score signature was constructed and we demonstrated its predictive ability through multi-cohorts. The low- and high-ICD_score groups are characterized by immune activation and suppression, respectively. Patients with different ICD_scores displayed heterogeneous clinicopathological characteristics, genetic features, response to treatment, as well as prognosis. Moreover, a significantly different immune landscape was observed in the two score groups. In the high-ICD_score group, the proportion of immunosuppressive cell infiltration was up-regulated and the immune checkpoint genes expression was enhanced. To further demonstrate these findings in the real world, we performed mIF on the pathological resections of one responder and one non-responder receiving immunotherapy and verified that patients with low ICD_scores comprised more anti-tumor immune cells. Finally, we built a quantitative nomogram model by incorporating the ICD_score, age, and TNM stage, which further enhanced the power and contributed to the clinical application of the ICD_score signature.

Despite the booming development and great potential of cancer immunotherapy, GC patients still show heterogeneity in their responses, indicating the necessity of developing synergistic therapeutic approaches to activate the tumor immune microenvironment, thereby promoting therapeutic efficacy. Recently, studies showed that inducing the ICD of tumor cells is a potentially effective strategy to elicit anti-tumor immunogenicity in support of immunotherapy [42]. Furthermore, oxaliplatin-based chemotherapy achieved greater efficacy because of additionally induced ICD [43]. 

The ICD was defined as a unique form of regulated cell death, which stimulates the anti-tumor immune system by exposing danger signals or DAMPs [3,5]. It has been demonstrated that ICD within the tumor microenvironment can be induced via chemotherapy, photothermal therapy (PTT), and radiotherapy (RT) [44]. Meanwhile, ICD that is driven by cancer therapy reshapes the TIME [45,46,47]. Mechanistically, ICD happens through DAMPs’ exposure or release, which contributes to DCs maturation and the infiltrating lymphocytes [48], thereby creating an immune-responsive tumor microenvironment [49]. Given the importance of ICD in tumorigenesis and progression, we attempted to stratify GC patients into different groups according to the expression of ICD-related genes to further explore the concrete role of these genes in the GC population. The ICD-related parameters involved in our analyses were identified by an extensive literature survey [7,27,28,29,30]. Interestingly, we identified three ICD-associated subgroups that exhibited significantly different TME landscape and immune statuses. 

Previous studies suggested that patients with a high proportion of tumor infiltration lymphocytes (TILs), PD-1 or PD-L1 expression are more likely to respond to immunotherapy. However, these biomarkers are applicable only partially to the responders in GC. Therefore, the construction of a predictive signature that can identify responders from non-responders to immunotherapy is urgently needed. To aid therapeutic decision-making, we developed an ICD_score signature with good performance in quantifying the prognostic risk. A high-ICD_score group showed an advanced TNM stage and a shorter predicted survival time. The landscape analysis of TIME and the data of mIF experiments verified that the ICD_score signature is valuable for immunotherapy. Patients with low ICD_scores possessed more activated immunocyte infiltration, which led to a better response to immunotherapy. The scoring signature is constructed by three genes (IL1A, NT5E, and TLR7), whose expression in the tumor is significantly higher than normal both in the TCGA cohort and our PKUCH cohort. CD73 (ecto-5′-nucleotidase; NT5E), one of the constructed signature genes, is a membrane-bound enzyme that catalyzes the transformation of AMP to adenosine. It is a crucial compound of inflammation that serves as an immunosuppressant to provide survival advantages to the tumor [50]. Several studies verified that NT5E is involved in oncogenesis, cancer immune escape, and therapeutic resistance [51,52,53]. IL1A encodes interleukin (IL)-1α, which is synthesized by monocytes and macrophages. As a member of the interleukin family, it undergoes proteolytical processing and is released companied by cell injury, allowing it to induce apoptosis [54]. The expression of IL-1α is up-regulated in tumor tissue compared to para-tumor tissues in several cancer types [55,56,57]. In response to infection, the Toll-like receptors promote inflammatory cytokine production by recognizing pathogen-associated and danger-associated molecular patterns [58]. It has been reported that targeted inhibition of TLR7 could strengthen the innate immune response in AML [59]. This scoring signature only compromised three representative high-risk genes, while the prediction accuracy did not decrease, which may be of great clinical application value in the future. 

Although our research showed advantages in multi-cohort verifications, there were still some limitations that require consideration. The signature presented here was established and validated using retrospective data; therefore, large-scale prospective clinical cohorts are needed to validate its clinical utility. More GC chemo-immunotherapy data need to be excavated to evaluate the synergistic effects of chemotherapy combined with immunotherapy induced by ICD. In the future, we will focus on the results of single-cell sequencing, which could help us further understand the heterogenicity in GC. 

## 5. Conclusions

Here, we comprehensively studied the roles of the ICD-related genes in GC for the first time and constructed an ICD_score signature with a good performance on assessing the prognosis of patients with GC and predicting treatment response. A nomogram based on the signature revealed a strong prognostic power for GC patients, which is worthy of clinical promotion. Consequently, the ICD_score signature could be applied as a novel reference to guide treatment and predict prognosis in GC patients.

## Data Availability

The original data presented in this study are included in the article or supplement materials, other information can be directed to the corresponding authors.

## Supplementary Materials

The following supporting information can be downloaded at: https://www.mdpi.com/article/10.3390/biom13030528/s1, Figure S1: The entire analytical process of this study; Figure S2: The relationship between PIK3CA mutation and the expression level of ICD-related genes in GC; Figure S3: Identification of the key module by WGCNA. (A) Clustering dendrogram of GC samples and heatmap of clinical traits. The clustering was based on the expression data of robust DEGs. The color intensity in heatmap increased with clinical traits. In terms of survival status, white means alive and red means dead. (B) Dendrogram of all candidate genes clustered based on a dissimilarity measure (1-TOM) together with assigned module colors. (C) Analysis of the scale-free fit index (left) and the mean connectivity (right) for various soft thresholding power values. (D–F) Module membership vs. gene significance in blue module. (G–I) Module membership vs. gene significance in brown module. (J) Venn diagram showing the overlap hub genes between two hub modules (module blue and brown). Hub gene restriction: GS > 0.5, MM > 0.8.; Figure S4: Univariate analysis evaluates the prognostic value of the hub genes plus fifty-three ICD genes in terms of OS; Figure S5: Validation of ICD_score in internal testing set and external validation set. (A) KM analysis of the OS between the two groups in testing set. (B) The ranked dot plot indicates the ICD_score distribution and scatter plot presents the patients’ survival status in testing set. (C) The PCA analysis demonstrated that the patients in the different ICD_score groups were distributed in two directions in testing set. (D–F) The same analysis was performed in external validation set and similar results were obtained; Figure S6: Comparison of the AUCs of the nomogram and age or TNM staging system. (A–C) AUCs of the nomogram, age and TNM stage to predict overall survival on Year 1 using the training set (A), internal testing set (B), and external validation set (C). (D–F) AUCs of the nomogram, age, and TNM stage to predict overall survival on Year 3 using the training set (D), internal testing set (E), and external validation set (F). (G–I) AUCs of the nomogram, age, and TNM stage to predict overall survival on Year 5 using the training set (G), internal testing set (H), and external validation set (I); Table S1: Summary of 53 recognized ICD-related genes; Table S2: prognostic analysis of ICD_related genes; Table S3: Clinical information of 443 gastric cancer patients; Table S4: Basic information of gastric cancer datasets included in the current study; Table S5: Identification of biological process on hub genes by clueGO.
