# Supplementary material for: Identification of Immunogenic Cell-Death-Related Subtypes and Development of a Prognostic Signature in Gastric Cancer
Source: Biomolecules. 2023 Mar 14;13(3):528. doi: 10.3390/biom13030528 (PMC10046021; doi:10.3390/biom13030528)
Supplement: Supplementary file 1 [file biomolecules-13-00528-s001.zip › Supplementary_Figures.pdf]

Supplementary Figures

Figure S1

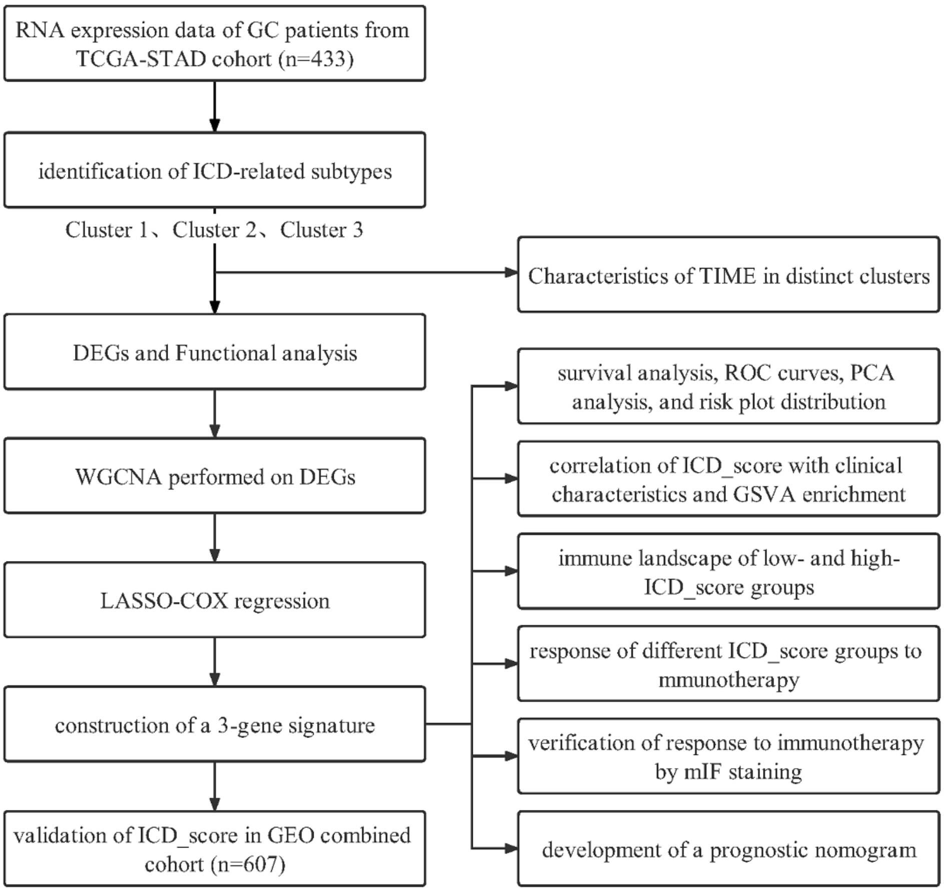

Figure S1. The entire analytical process of this study.

Figure S2

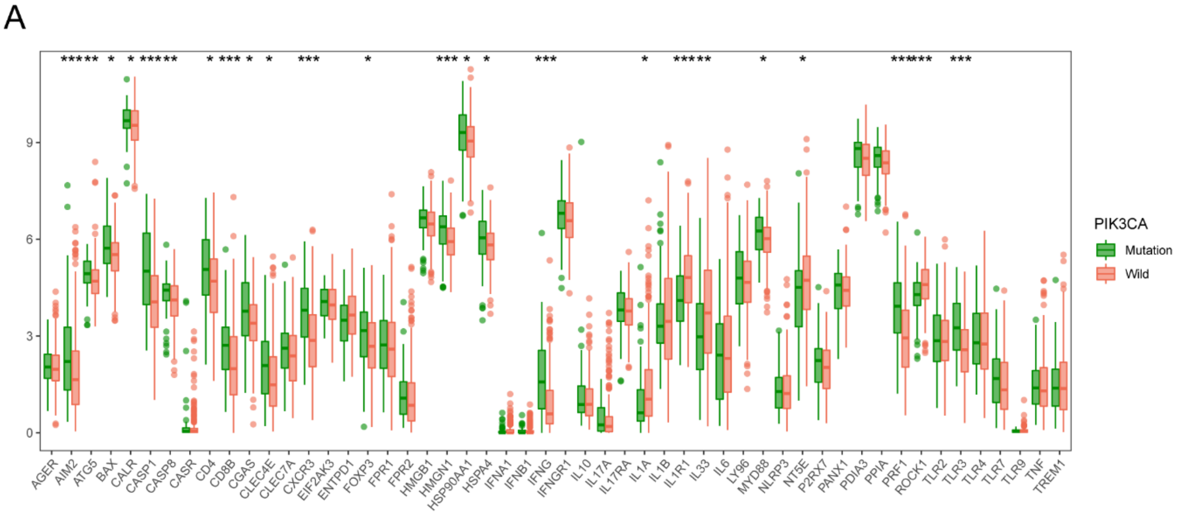

Figure S2 The relationship between PIK3CA mutation and expression level of ICD-related genes in GC.

Figure S3

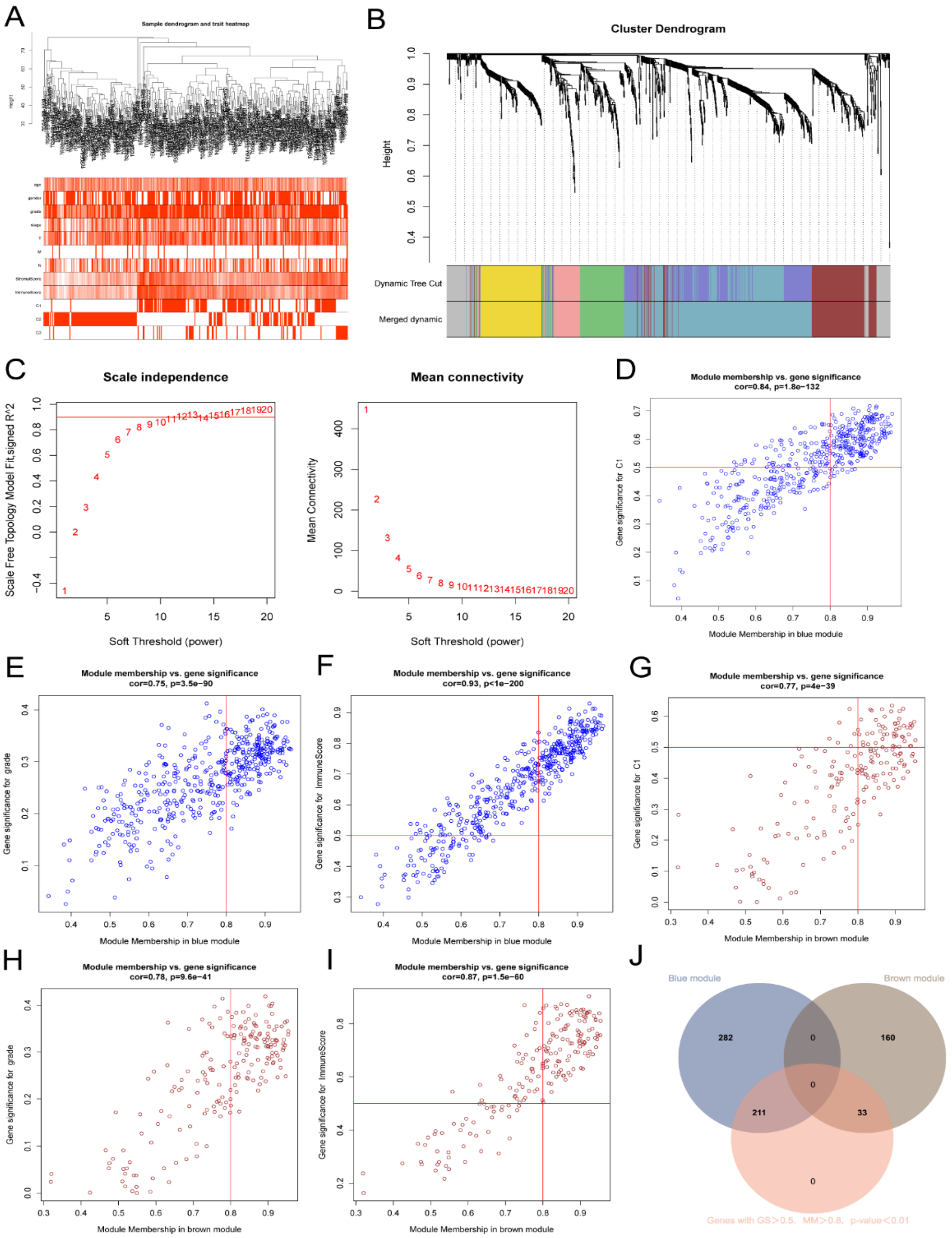

Figure S3 Identification of the key module by WGCNA. (A) Clustering dendrogram of GC samples and heatmap of clinical traits. The clustering was based on the expression data of robust DEGs. The color intensity in heatmap increased with clinical traits. In terms of survival status, white means alive and red means dead. (B) Dendrogram of all candidate genes clustered based on a dissimilarity measure (1-TOM) together with assigned module colors. (C) Analysis of the scale-free fit index

(left) and the mean connectivity (right) for various soft-thresholding power value. (D-F) Module membership vs. Gene Significance in blue module. (G-I) Module membership vs. Gene Significance in brown module. (J) Venn diagram showing the overlap hub genes between two hub modules (module blue and brown). Hub genes restriction: GS > 0.5, MM > 0.8.

Figure S4

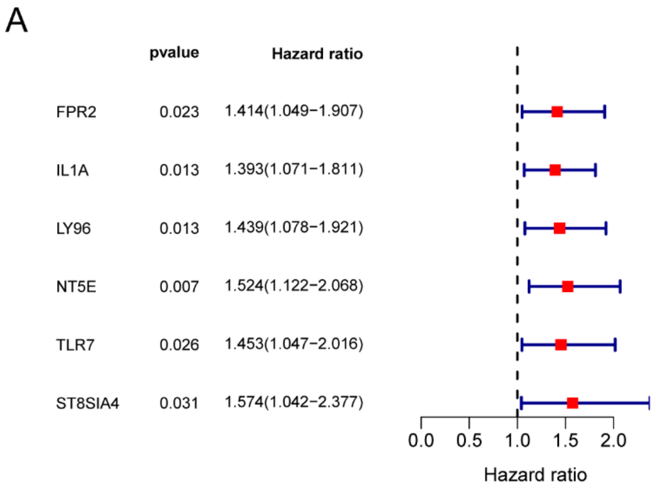

Figure S4 Univariate analysis evaluates the prognostic value of the hub genes plus fifty-three ICD genes in terms of OS.

Figure S5

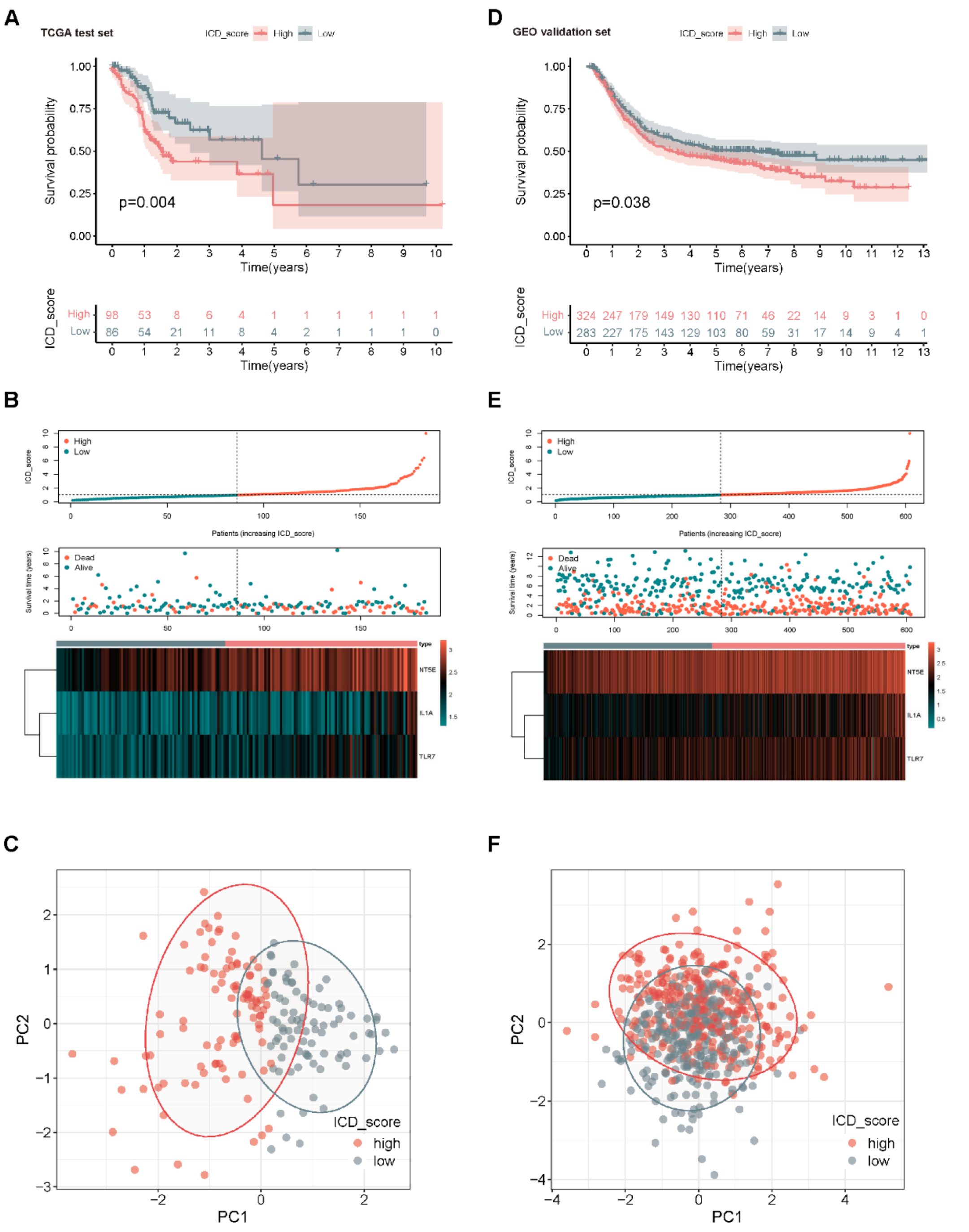

Figure S5 Validation of ICD_score in internal testing set and external validation set. (A) KM analysis of the OS between the two groups in testing set. (B) The ranked dot plot indicates the ICD_score distribution and scatter plot presenting the patients' survival status in testing set. (C) The PCA analysis demonstrated that the patients in the different ICD_score groups were distributed in two directions in testing set. (D-F) The same analysis was performed in external validation set and similar results were obtained.

Figure S6

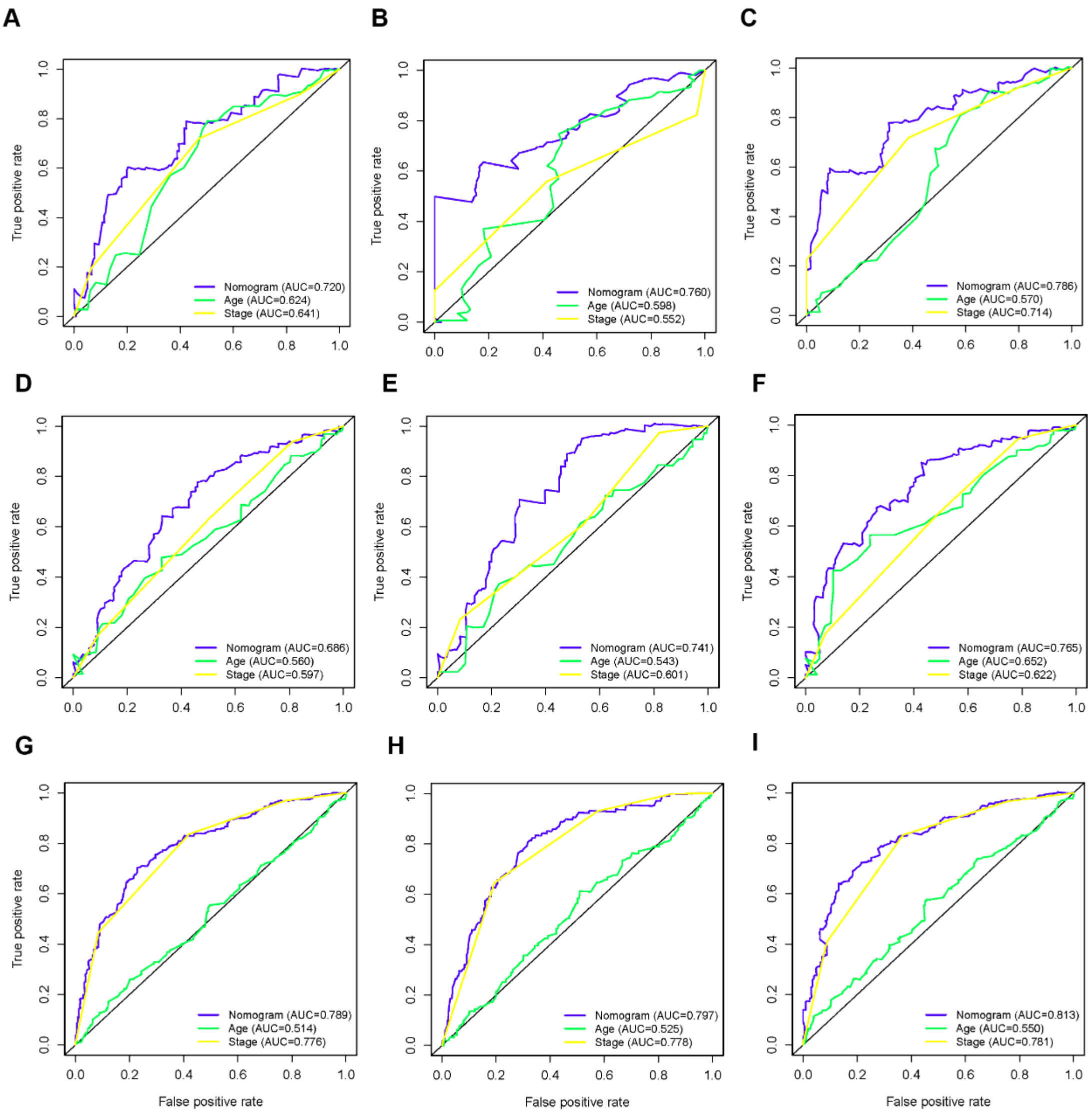

Figure S6 Comparison of the AUCs of the nomogram and age or TNM staging system. (A-C) AUCs of the nomogram, age and TNM stage to predict overall survival at 1-year using the training set (A), internal testing set (B) and external validation set (C). (D-F) AUCs of the nomogram, age and TNM stage to predict overall survival at 3-year using the training set (D), internal testing set (E) and external validation set (F). (G-I) AUCs of the nomogram, age and TNM stage to predict overall survival at 5-year using the training set (G), internal testing set (H) and external validation set (I).
